# Task-Similarity-Based VNF Aggregation for Air–Ground Integrated Networks

**DOI:** 10.3390/s23042259

**Published:** 2023-02-17

**Authors:** Mingfeng Chen, Qiyong Chen, Zhaoyu Su, Shaohua Sun, Chunhai Li

**Affiliations:** 1 School of Artificial Intelligence, Guilin University of Electronic Technology, Guilin 541004, China; 2 School of Information and Communication, Guilin University of Electronic Technology, Guilin 541004, China

**Keywords:** air–ground network, service function chaining, improved genetic algorithm, function aggregation

## Abstract

In a harsh environment, function aggregation of air–ground integrated network service function chaining (SFC) deployment can easily cause network load imbalance, which affects the network security and reliability. In this study, a task-similarity-based virtual network function (VNF) aggregation scheme was proposed. It considered air–ground network resource consumption and load balance before SFC mapping. A model for selecting VNFs to be aggregated based on task similarity was built. The tasks were classified based on their similarity. Furthermore, the VNFs to be aggregated were selected within the class under the constraints of the underlying physical resources. Load balancing was achieved by adjusting the similarity threshold. Moreover, an SFC mapping selection scheme based on network resource awareness was used to obtain the most suitable physical nodes for single-chain and multi-chain mapping according to various attributes of physical network nodes. The simulation results indicated that the proposed scheme with a better load balance design outperformed existing works on VNF aggregation. We also demonstrated that the task-similarity-based scheme was resource-consumption efficient and effective.

## 1. Introduction

The air–ground integrated network makes up for the shortcomings of the independent ground network and the air network composed of drones. Network integration provides seamless global connections and highspeed computing capabilities [1] to satisfy the service quality requirements of some harsh environments, such as those experienced when searching forests, doing reconnaissance, jamming and surveying trenches. However, the rapid deployment of new tasks has led to a rapid increase in the amount of hardware and caused further problems such as high operating costs, insufficient resource utilization and inflexible management [2]. Network function virtualization (NFV) reduces the operating cost and improves the reliability of network deployment [3] by decoupling hardware devices and the network functions running on them [4].

In the NFV network, the required functional requirements are fulfilled by instantiating the virtual network function (VNF). To improve resource utilization, different studies adopted different function aggregation methods. For example, in [5], the VNF that first appeared in the SFC in the terrestrial–satellite hybrid cloud network was used to obtain its optimal deployment position using linear planning. When the same VNF appeared in the subsequent service, the requested SFC was merged with the SFC with the same VNF running in the cloud and processed as new resource consumption. If the mapping is successful, the merge will deploy to the new cloud for processing. Otherwise, the optimal deployment plan obtained according to linear planning will be directly deployed in the cloud. In [6], the concept of an aggregation rate was proposed in the air–space–ground integration network for service reconfiguration based on the service function chain. It was expressed as the ratio of the number of instantiated VNFs to the total number of VNFs that are required by all service requests. The instantiated VNFs were expressed as the difference between the number of VNFs required and the number of VNFs deployed. Then, all the different types of VNFs were gathered together to obtain the overall degree of aggregation by adjusting the aggregation rate. In [7], the SFC mapping problem was transformed into a VNF integration problem and a VNF combined reuse method based on the greedy algorithm was designed to reduce the number of virtual function instances.

However, due to the difference between the arrival time and the life cycle of the SFC request in the NFV network, such a functional aggregation will cause some physical nodes to deploy a large number of VNFs. When the resource occupancy rate reaches the bottleneck, the quality of service (QoS) will drop sharply [8]. At the same time, there are a large number of nodes that only deploy a small number of VNFs in the network, and many resources are idle. If the VNF can be reasonably aggregated, the physical network load balance can be realized and the QoS can be improved while entirely using the underlying network resources.

So far, there have been related works on the network load balancing problem of a single network under NFV technology, but there are few studies on load balancing in an air–ground integrated network. In [9], load balancing in a satellite network was considered after virtual mapping. The VNF of the overloaded node was migrated to other suitable nodes, and the load balancing was realized by reconfiguring the service function chain. In [10], a new service chain reconstruction architecture was proposed to achieve load balancing in the NFV environment. When processing the current service chain, the mapping of the service function chain followed the path optimization principle and was realized by transferring other instantiated VNFs when it encountered a conflict. In [11], load balancing was achieved by using a virtual SDN controller as a VNF, but adding an SDN controller increased the operator’s overhead. In [12], a fast adaptive migration algorithm for a VNF based on multi-dimensional environment awareness was proposed by using fixed-threshold resource awareness to calculate the VNF to be migrated. It used the technique for order preference by similarity to ideal solution (TOPSIS) algorithm to evaluate nodes and select the migration destination node. Based on ensuring the migration cost, the load balancing of the underlying network was improved, but the ratio of revenue to cost after the migration was not considered. In [13], a VNF migration method that optimized the network impact and whose priority goal was to reduce the SFC delay was proposed. At the same time, it took into account the node load and migration cost, effectively improving the timeliness of SFC. However, the load balancing of the network was not significantly improved. Although these studies solved the problem of load imbalance to a certain extent, most of them are realized by restructuring the VNF migration of the service function chain. Still, it is easy to cause the link to be interrupted and it is difficult to guarantee the service quality requirements in harsh environments.

Due to the apparent similar characteristics of task types in the air–ground integrated network, this study proposed a VNF aggregation method based on task similarity and network resource awareness that considered network load balancing before SFC mapping. First, a model for selecting the VNFs to be aggregated based on task similarity was proposed. The tasks were classified based on their similarity, the functions to be aggregated were selected within the class under the constraints of the underlying physical resources and the load balance was achieved by adjusting the similarity threshold. Second, a resource awareness SFC mapping node selection algorithm was used to obtain the most suitable physical node for single-chain and multi-chain mapping according to the various attributes of physical network nodes. It can make full use of the underlying physical resources to reduce resource consumption and load imbalance.

## 2. The System Model

Since the air–ground integrated network and the service function chaining have node attribution and link attribution that are remarkably similar to those of graphs in the mathematical model, they are modeled as different types of graphs according to their characteristics. Due to the diversity and heterogeneity of air–ground network resources, it is necessary to distinguish them according to their attribution when modeling.

### 2.1. Model Building

**Air–ground physical network:** We regarded the air–ground physical network as a strongly connected directed graph, which is represented by tuples W=<N,A>, where N is the set of network nodes and A represents the set of physical links. The rich resources of the ground network can solve more complex tasks, but the base station is fixed and the coverage is limited. The air network composed of UAVs covers a wide range, but computing resources are scarce. In the air–ground integrated network, the most appropriate forwarding strategy can be selected according to the service requirements to flexibly access the air or ground network to meet the business requirements. Therefore, the resources and coverage of nodes in air–ground physical networks, namely, network connectivity and computing capacity, are different. Nodes in different networks need to be distinguished. In an air–ground network, physical network nodes are represented by $N=N_{S}\cup N_{G}$, where $N_{S}$ is the set of air network nodes and $N_{G}$ is the set of ground network nodes. Similarly, physical links are represented by $A=A_{S}\cup A_{G}\cup A_{C}$, where $A_{S}$ is the spatial link set, $A_{G}$ is the ground link set and $A_{C}$ is the air–ground link set. {(a,b)∈A|a,b∈N} is used to represent all routing paths from the initial node a to endpoint b. Each node has the ability to handle specific functions, but each node can only handle one function at a time, and each physical node has fixed computing power $\left\{C_{n}|C_{n}>0.n\in N\right\}$.$\left\{D_{a,b}|D_{a,b}>0,(a,b)\in A\right\}$ and $\left\{B_{a,b}|B_{a,b}>0,(a,b)\in A\right\}$ represent the bandwidth consumption and latency consumption of the physical link, respectively.

**Service request definition:** We treated the service request as a directed acyclic graph, assuming that a batch of requests is processed with an interval T. Of course, these requests may arrive at any time during the last processing interval. This study focused on batch processing within the time interval T. Service request R is represented by tuples R=<F,E>, where R={r|r=1,2,…,|R|} represents the service request that arrives within time T. The source and destination nodes of each service request are represented by $\left\{s_{r}|r\in R\right\}$ and $\left\{d_{r}|r\in R\right\}$, respectively. The node of the service request is the VNF, and the link is the dependency among the VNFs. There are k types of VNFs required by the service, including bandwidth requirements $\left\{B_{r}|r\in R\right\}$, computing power requirements $\left\{C_{r}|C_{r}>0.r\in R\right\}$ and the delay deadline $\left\{D_{r}|r\in R\right\}$. Each service request r consists of a set of VNFs $\left\{f_{1},f_{2}\ldots f_{i}|i\in F\right\}$, and the link $E_{r}=\{(p,q)|p,q\in F,r\in R\}$ between the VNFs needs to follow the dependencies between virtual network functions.

**Consumption model:** To visually express the SFC mapping and to make calculations more convenient, the model variables were defined in matrix form. The mapping of unknown vertices $X_{\psi }$ and the mapping of links $Y_{\psi }$ were also represented by a binary matrix. Define $x_{f,a}^{r}$ as a virtual network function deployment variable for task r, and when its value is 1, this means that the virtual network function is deployed on the physical node a. $\prod =\{\prod_{ab}\},\forall a,b\in N$ represents the collection of all paths in the network nodes. Define the virtual link mapping variable $y_{pq,ab}^{r}$, which has a value of 1 when the link between two adjacent virtual network functions p and q in task r is mapped to the underlying physical link $\prod_{ab}$; otherwise, $y_{pq,ab}^{r}$ has the value 0.

We defined a virtual network function mapping binary matrix X for all possible vertex mappings.

(1) $$x_{f,a}^{r}=\left\{\begin{array}{c} 1,\;if\;X_{\psi }(f)=a\\ 0,\;otherwise \end{array}\right.$$

Similarly, we defined a binary matrix Y of link mappings that represents all possible link mappings.

(2) $$|E|\cdot |\prod |=\frac{\left|F|\cdot (|F|-1\right)}{2}\cdot |N{|}^{2}$$

(3) $$y_{pq,ab}^{r}\left\{\begin{array}{c} =1,\;if\;Y_{\psi }(C_{pq})=\prod_{ab}\\ =0,otherwise \end{array}\right.$$

|E|⋅|∏| is the binary matrix Y of all possible link mappings.

For each successfully deployed SFC, the following constraints must be met:

**Uniqueness constraint:** a virtual network function of a task can only be embedded on one physical node, and a virtual link of a task can only be mapped to a unique physical link.

(4) $$(\forall f\in F)\sum_{a\in N}x^{r}{}_{f,a}=1$$

(5) $$(\forall E_{pq}\in E)\sum_{\prod_{ab}\in \prod }y_{pq,ab}^{r}=1$$

**Bandwidth constraint:** each path in the routing path must have enough bandwidth to meet the requirements of the virtual link.

(6) $$y_{pq,ab}^{r}\cdot B_{b,a}<B_{r}$$

**Computing power constraint:** the physical node needs to have enough computing resources to handle the data traffic carried by the SFC.

(7) $$C_{f}\cdot x_{f,a}^{r}<C_{a}$$

**Compatibility constraints:** Each virtual network function can only be mapped to a physical node capable of processing this function, and a virtual link can only be mapped to a physical link with sufficient bandwidth resources, that is, the bandwidth resources of the physical link must meet the bandwidth resources required by the service request.

(8) $$(\forall f\in F,\forall a\in N)x_{f,a}^{r}=\left\{\begin{array}{c} 1,\;if\;type(f)=type(a)\\ 0,\;otherwise \end{array}\right.$$

(9) $$\forall E_{pd}\in E,\forall A_{ac}\in A)y_{pd,ac}^{r}=\left\{\begin{array}{c} 1,B_{pd}\leq B_{ac}\\ 0,otherwise \end{array}\right.$$

The function type determines the type of VNF and can only be mapped when the type of VNF and the type carried by the physical node are the same.

We used the load-balancing index α to measure the effect of network load balancing, where the formula is

(10) $$\alpha =\sum_{k}\sum_{n\in N}{\left(\omega_{k}^{n}-\bar{\omega }\right)}^{2}$$

where $\omega_{k}^{n}$ represents the k-type resource occupancy rate of node n and $\bar{\omega }$ represents the average k-type resource occupancy rate of each physical node.

Since the computational consumption of the processing VNF is fixed, the resource consumption requested by the task in this article includes instantiation consumption and bandwidth consumption. However, air–ground integrated network nodes are different in terms of coverage and computing power. Therefore, their instantiation overhead and bandwidth consumption are different.

(11) $$\begin{array}{ll} \cos t(r) & =\sum_{r\in R}(\underset{\mathrm{Air}\;\mathrm{node}\;\mathrm{instantiation}\;\mathrm{overhead}}{\underbrace{\sum_{a\in N_{S}}(\gamma_{f,a}\cdot x^{r}{}_{a,f})}}+\underset{\mathrm{Ground}\;\mathrm{node}\;\mathrm{instantiation}\;\mathrm{overhead}}{\underbrace{\sum_{a\in N_{G}}(\varepsilon_{f,a}\cdot x^{r}{}_{a,f})}}\\ & +\underset{\mathrm{Air}\;\mathrm{link}\;\mathrm{bandwidth}\;\mathrm{consumption}}{\underbrace{\sum_{{}_{a,b}\in A_{S}}(D_{pq,ab}\cdot y^{r}{}_{pq,ab})}}+\underset{\mathrm{Ground}\;\mathrm{link}\;\mathrm{bandwidth}\;\mathrm{consumption}}{\underbrace{\sum_{{}_{a,b}\in A_{G}}(B_{pq,ab}\cdot y^{r}{}_{pq,ab})}}\\ & +\underset{\mathrm{Air}-\mathrm{Ground}\;\mathrm{link}\;\mathrm{bandwidth}\;\mathrm{consumption}}{\underbrace{\sum_{{}_{a,b}\in A_{C}}(Q_{pq,ab}\cdot y^{r}{}_{pq,ab})}} \end{array}$$

$\gamma_{f,a}$ represents the instantiation overhead of mapping function f to physical node a in the air network, and $\varepsilon_{f,a}$ represents the instantiation overhead of mapping function f to physical node a in the ground network. $D_{pq,ab}$ represents the actual bandwidth requirement for mapping virtual link pq to physical link ab in the air network, $B_{pq,ab}$ represents the actual bandwidth requirement for mapping virtual link pq to physical link ab in the ground network and $Q_{pq,ab}$ represents the actual bandwidth requirement for mapping virtual link pq to physical link ab in the air–ground links.

### 2.2. Selection Method of VNFs to Be Aggregated Based on Task Similarity

If each VNF is instantiated for each SFC, it will cause a huge instantiation overhead and waste of resources when deploying the service function chain. VNF aggregation effectively solves this problem. However, the existing function aggregation methods only focus on reducing the instantiation overhead and ignoring the load balancing problem caused by VNF aggregation. To solve the problem of load balance, Refs. [9,10,12,13] adopted VNF migration and solved it using SFC reconstruction, which seriously affected the stability of the link.

**Figure 1:**
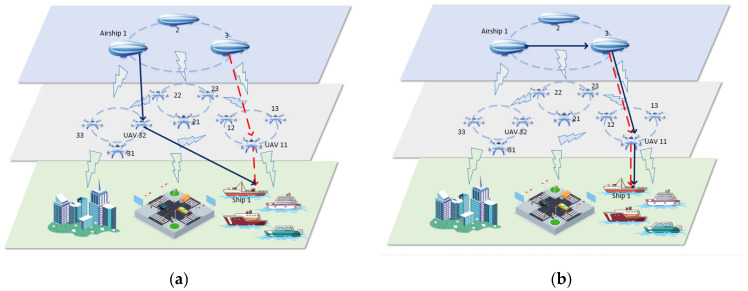
Task processing (**a**) without task similarity and (**b**) with task similarity.

As shown in Figure 1a, when airship 3 communicates with ship 1, the link indicated by the dashed line is selected to complete the communication. When airship 1 also receives the task of communicating with ship 1, due to the independence of the tasks, it chooses the link represented by the solid line to communicate. This not only causes a waste of network resources but also makes the links between nodes repeatedly disconnected, causing a network server instability. If the routing path of airship 1 can be shown as the solid line linked in Figure 1b according to the similarity of the tasks, this problem will be solved.

In response to the above problems, this study proposed a VNF selection method to aggregate tasks based on task similarity.

The core idea of the method is as follows:

(1) For tasks that arrive in time interval T, the virtual network functions required by the task and the access to task resources that are more similar are placed into the same category. Tasks within the same class share the same type of physical node.
(2) In the same category, the same VNF is selected for functional aggregation under the condition of satisfying bandwidth and computing constraints.
(3) By adjusting the similarity threshold, there is a trade-off between resource consumption and load balancing.

The specific implementation is as follows:

Task similarity is divided into functional similarity and resource similarity. Task similarity within the time interval T is considered in order to divide similar tasks into the same class to share physical network nodes with the same functionality. On the one hand, this can reduce the instantiation overhead of the same virtual network function for similar tasks, reduce the global waiting time and maintain network stability. On the other hand, it can reduce the uneven load caused by all tasks sharing the same physical node for the same virtual network function. However, the task similarity division according to the time interval T cannot completely solve the problem of an uneven load, and thus, the task similarity threshold can be adjusted to balance the resource consumption and load balancing to meet the task requirements in different scenarios.

The definition of task similarity in the time interval T is given here, which includes virtual network function similarity and resource similarity.

Virtual network function similarity refers to the similarity of VNFs by different tasks.

(12) $$precent_{r_{i}}=\frac{typequal(r_{i,}r_{j})}{totaltype(r_{i})}$$

(13) $$Tsim(r_{i},r_{j})=\frac{precent_{r_{i}}+precent_{r_{j}}}{2}$$

$Tsim(r_{i},r_{j})$ is the similarity of the virtual network functions of the two tasks $r_{i}$ and $r_{j}$. $typequal(r_{i,}r_{j})$ is the number of virtual network functions of the same type contained in tasks $r_{i}$ and $r_{j}$. $totaltype(r_{i})$ is the total number of virtual network functions in service request $r_{i}$ and $precent_{i}$ is the proportion of the number of virtual network functions of the same virtual network functions in task $r_{i}$ to the total number of virtual network functions.

When t tasks arrive, the system first calculates the similarity of the pairwise tasks and then divides it by the number of pairwise permutations and combinations to obtain the task similarity between t tasks.

When t tasks arrive, the virtual network function similarity of the tasks is

(14) $$Tsim(r_{i},r_{j},\ldots r_{t})=\frac{\sum_{i,j\in t(i\neq j,i<j)}Tsim(r_{i},r_{j})}{C_{t}^{2}}$$

Resource similarity refers to the similarity of the actual demand for the same virtual network function resources.

(15) $$r_{f_{i}}=\{v_{1},v_{2},\ldots v_{l}|l\in k\}$$

where $r_{f_{i}}$ represents the resources that task r contains for virtual network function $f_{i}$ that needs to be accessed.

(16) $$Ssim(f^{i},f^{j})=(\sum_{i=1}^{m}\frac{\sum_{l=1}^{k}\omega_{il}\times \omega_{jl}}{\sqrt{(\sum_{l=1}^{k}\omega_{il}{}^{2})+(\sum_{l=1}^{k}\omega_{kl}{}^{2})}})/m$$

$Ssim(f^{i},f^{j})$ represents the similarity of resources required for the same virtual network function in tasks $r_{i}$ and $r_{j}$. $\omega_{il}$ and $\omega_{il}$ are the actual demand for the lth resource of the same virtual network function f for tasks $r_{i}$ and $r_{j}$, respectively.

When t tasks arrive, the resource similarity is

(17) $$Ssim(f^{i},f^{j},\ldots f^{m})=\frac{\sum_{i,j\in m(i\neq j,i<j)}Ssim(f^{i},f^{j})}{C_{m}^{2}}$$

The task similarity is

(18) $$Sim(r_{i},r_{j},\ldots r_{t})=\frac{Tsim(r_{i},r_{j},\ldots r_{t})\times (\gamma +\xi \times Ssim(f^{i},f^{j},\ldots f^{m}))}{\gamma +\xi }$$

where γ and ξ are weights.

After classification according to task similarity, the system judges whether the aggregation can be performed by whether the bandwidth and computing resources meet the physical network constraints. The aggregation can be performed when the constraints are met; otherwise, it is achieved by adjusting the similarity. Each time, the network resource status is updated after a division by task similarity. For load balancing, this study took an approach to determine task classification by presetting similarity thresholds. While ensuring that the resource consumption is reduced, load balancing is achieved by adjusting the similarity threshold to avoid too many tasks waiting for the same physical node to meet the requirements of the tasks.

## 3. SFC Mapping Node Selection Algorithm Based on Resource Awareness

In order to complete the user’s service request, after the task that arrives in time T is classified by task similarity, the task is deployed on the appropriate physical node through the SFC mapping algorithm. The algorithm we proposed was based on the awareness of network resources, which includes the remaining resources of the link bandwidth and the usage of computing resources of nodes. We adopt an improved genetic algorithm to select the mapped physical network node through the awareness of network resources. Different SFC construction schemes will cause different mapping results of the service function chain, and different mapping results will also be obtained by selecting different physical nodes for mapping. Compared with searching for a single VNF separately or mapping using graph theory, the feature of genetic algorithm exploring from a string set reduces the complexity of the algorithm. At the same time, a genetic algorithm can easily realize parallelization. It can handle multiple individuals in the group and simultaneously deal with different construction schemes of SFC. However, the efficiency of a single genetic algorithm is low, and it can easily converge prematurely. A particle swarm has the advantages of fast calculation speed and solid global searchability; therefore, this study proposed a genetic-particle swarm optimization (GA-PSO) algorithm.

The particle swarm algorithm is added to the genetic algorithm as the genetic parent chromosome. The speed and position update of the particle swarm is replaced by the crossover and mutation of the genetic algorithm. For single-chain and multi-chain deployments, different coding, crossover and mutation methods are used to implement the deployment of service function chains.

(1) Chromosome coding

**Figure 2:**
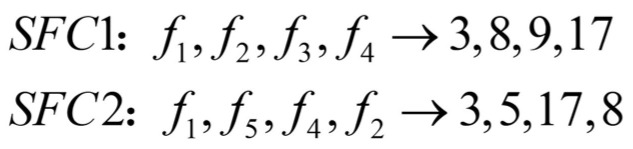
Single-chain individual coding.

**Single-chain individual mapping coding:** When performing single-chain mapping, SFCs in the same category are deployed one after the other in the order of arrival. That is, within the same category, for the VNF that has been instantiated, the same VNF of the subsequent SFC is deployed on the same physical node, as shown in Figure 2. For the service function chain that satisfies the dependency, the shortest path method is used to calculate the path according to the service function chain mapping code.

**Figure 3:**
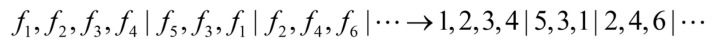
Multi-chain mapping and multi-encoding.

**Multi-chain and multi-encoding:** In the case of multi-chain mapping, SFCs in the same class are deployed simultaneously, multiple service function chains are coded as a chromosome and multiple SFC mapping schemes in the class are multi-encoded. The same VNF in the same category is mapped on the same physical node, as shown in Figure 3. The shortest path method is used to calculate the shortest path according to the deployment location of the aggregation function.

(2) Fitness function

**Single-chain:** When performing single-chain mapping, SFCs in the same category are deployed one after the other in the reference order. After each service function chain is deployed, the subsequent service function chains realize function sharing for the same VNF. Therefore, when deploying a single chain, the resource consumption needs to remove the overhead saved after aggregation. It includes the reduced instantiation overhead of the air nodes and ground nodes.

(19) $$\cos ts(r)=\cos t(r)-\underset{\begin{array}{l} \mathrm{reduced}\;\mathrm{instantiation}\;\\ \mathrm{overhead}\;\mathrm{of}\;\mathrm{air}\;\mathrm{nodes} \end{array}}{\underbrace{\sum_{a\in N_{S},f\in F}\gamma_{f,a}\cdot \psi_{f}}}-\underset{\begin{array}{l} \;\mathrm{reduced}\;\mathrm{instantiation}\;\\ \mathrm{overhead}\;\mathrm{of}\;\mathrm{ground}\;\mathrm{nodes} \end{array}}{\underbrace{\sum_{a\in N_{G},f\in F}\varepsilon_{f,a}\cdot \varphi_{f}}}$$

$\psi_{f}$ and $\varphi_{f}$ represent the number of instantiations that are reduced by the spatial and ground nodes after the function sharing function f.

**Multi-chain:** In multi-chain deployment, SFCs in the same category are deployed simultaneously. First, we select the optimal deployment location of the aggregated VNF and then find the deployment location of other VNFs. Therefore, when multiple chains are deployed simultaneously, the changes in bandwidth resources after aggregation should be considered. At the same time, the resource consumption needs to be subtracted from the overhead saved after aggregation, which includes the reduced instantiation overhead of the air nodes and ground nodes.

(20) $$\begin{array}{ll} \cos ts(r) & =\cos t(r)-\underset{\begin{array}{l} \mathrm{reduced}\;\mathrm{instantiation}\;\\ \mathrm{overhead}\;\mathrm{of}\;\mathrm{air}\;\mathrm{nodes} \end{array}}{\underbrace{\sum_{a\in N_{S},f\in F}\gamma_{f,a}\cdot \psi_{f}}}-\underset{\begin{array}{l} \;\mathrm{reduced}\;\mathrm{instantiation}\;\\ \mathrm{overhead}\;\mathrm{of}\;\mathrm{ground}\;\mathrm{nodes} \end{array}}{\underbrace{\sum_{a\in N_{G},f\in F}\varepsilon_{f,a}\cdot \varphi_{f}}}\\ & +\underset{\begin{array}{l} \;\mathrm{Increased}\;\mathrm{air}\;\mathrm{links}\;\\ \mathrm{bandwidth}\;\mathrm{consumption} \end{array}}{\underbrace{\sum_{{}_{a,b}\in A_{S}}B_{ab,pq}\cdot \varpi_{pq,ab}}}+\underset{\begin{array}{l} \mathrm{Increased}\;\mathrm{ground}\;\mathrm{links}\;\\ \mathrm{bandwidth}\;\mathrm{consumption} \end{array}}{\underbrace{\sum_{{}_{a,b}\in A_{G}}B_{ab,pq}\cdot \varpi_{pq,ab}}}+\underset{\begin{array}{l} \mathrm{Increased}\;\mathrm{air}-\mathrm{ground}\;\mathrm{links}\;\\ \mathrm{bandwidth}\;\mathrm{consumption} \end{array}}{\underbrace{\sum_{{}_{a,b}\in A_{C}}B_{ab,pq}\cdot \varpi_{pq,ab}}} \end{array}$$

(3) Chromosome crossover and mutation

**The chromosome crossover was divided into two parts**, namely, optimal crossover and free crossover. The optimal crossover is the crossover between the optimal chromosome and the common chromosome. It includes not only the crossover between the individual optimal chromosome and the common chromosome but also the crossover between the group optimal chromosome and the common chromosome. Free crossover avoids the fast convergence of the algorithm and crossovers the chromosomes obtained by the optimal crossover with common chromosomes. When the crossover is performed, the crossover bit is randomly selected for replacement. In the crossover operation, it is necessary to satisfy the conflict detection, that is, to satisfy the uniqueness constraint and the compatibility constraint.

When a chromosome is mutated, the mutation position is randomly selected. Then, a node is randomly selected from the set of physical nodes carrying the same VNF and is replaced at the mutated location.

When performing the crossover and mutation operations, it is necessary to satisfy the conflict detection, that is, to satisfy the uniqueness constraint and the compatibility constraint.

## 4. Simulation

### 4.1. Experimental Setup

To verify the performance of the algorithm, we used MATLAB 2018a software to simulate it on a computer, which was configured with an 8 GB Intel Core i5-4210U CPU. The simulation used the network topology on SNDlib [14], including 25 points and 45 edges. The point with the most robust connectivity was selected as the UAV space network node, and the other nodes were used as the ground network topology. In the experiment, the direction of the topological edge was undirected, and the bandwidth was 500 Mb/s. Five VNF types were selected in the experiment, and each node randomly selected two to three types as the VNF types that the node can bear. Each service request contained at least three VNFs and at most five VNFs. The minimum similarity of the tasks was 0.3, and the source node and destination node of the task were randomly selected.

### 4.2. Experimental Analysis

First, in order to verify the effectiveness and convergence of the GA-PSO algorithm proposed in this study, we randomly selected the task request and set the task request bandwidth to 20 M/s, the population number to 20, the number of iterations to 100 and an average of 200 experimental times. The results are shown in Figure 2.

**Figure 4:**
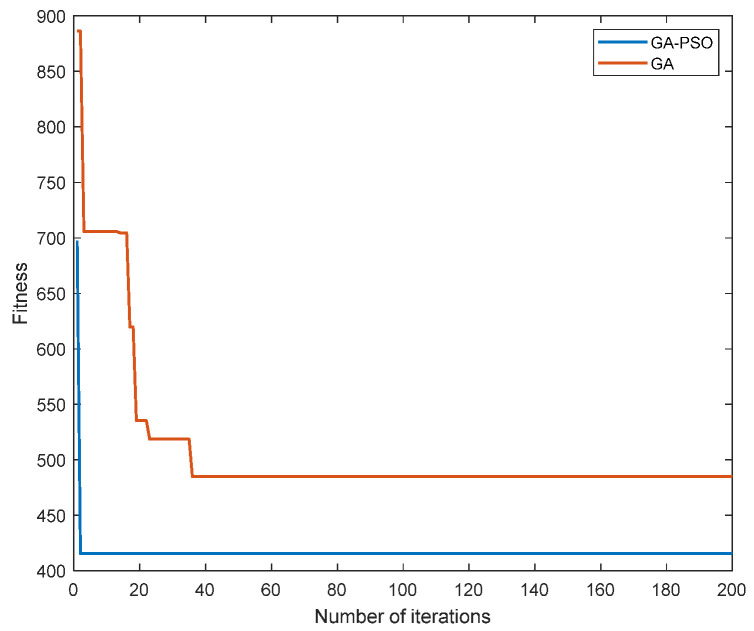
Comparison of the algorithm convergences.

It can be seen from Figure 4 that the GA-PSO algorithm proposed in this study was superior to the GA algorithm in terms of the convergence speed and fitness value.

**Figure 5:**
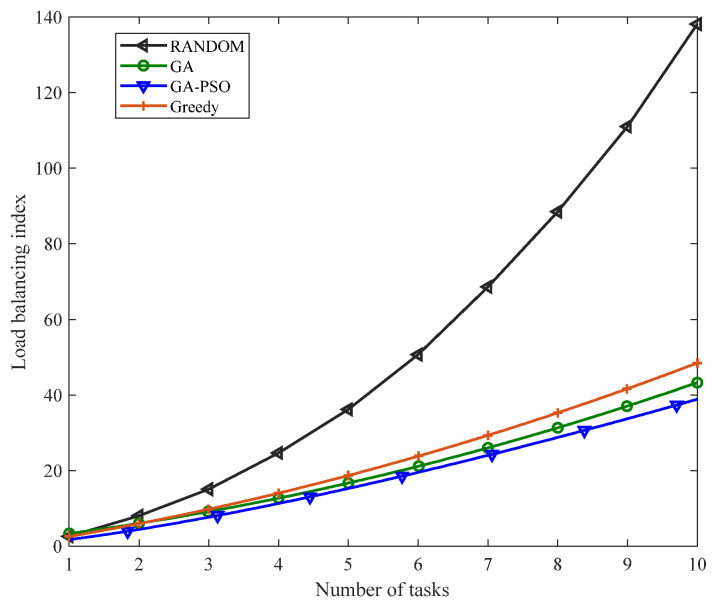
Comparison of the load-balancing indexes of different algorithms.

**Figure 6:**
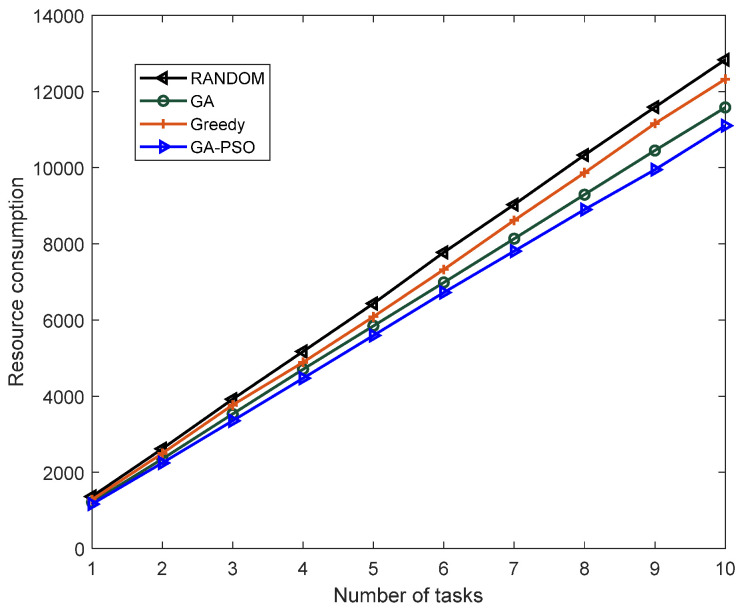
Comparison of the resource consumption of different algorithms.

Figure 5 and Figure 6 compare the load-balancing index and resource consumption of the proposed algorithm with the GA algorithm, greedy algorithm and RANDOM algorithm when given different tasks. As can be seen from Figure 5 and Figure 6, with the gradual increase in the number of tasks, this study proposed that the performance of the algorithm should always be better than other algorithms. This is because the RANDOM algorithm does not have any optimization strategy, but only randomly deploys SFC. The greedy algorithm only focuses on finding the optimal solution to the current subproblem, that is, only focusing on the location of the next virtual network function deployment, not on the consumption and load of the overall SFC deployment. The traditional GA algorithm has the defect of converging too fast and can easily fall into a local optimum, while the algorithm proposed in this study adds a particle swarm algorithm to the traditional genetic algorithm and improves the method of cross-mutation to give it better convergence and optimization; therefore, whether the system is load balancing or managing resource consumption, the performance of this study’s algorithm presents certain advantages.

**Figure 7:**
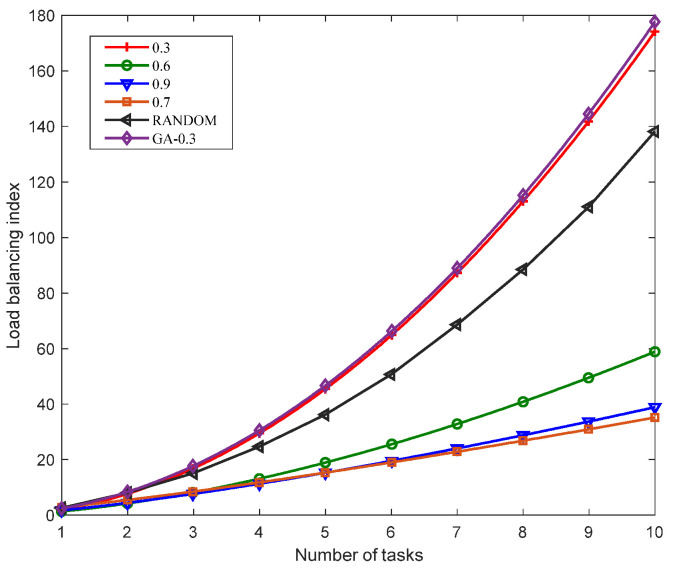
Comparison of the load-balancing indexes with different similarity thresholds.

Figure 7 shows the load-balancing index comparison of the single-chain deployment method proposed in this study with the different similarity thresholds, the GA algorithm with a similarity threshold of 0.3 and the randomly deployed RANDOM algorithm given the different number of tasks. Since the minimum similarity between tasks in this study was 0.3, when the similarity threshold was 0.3, all the same VNFs were aggregated, that is, the traditional aggregation method was used. Compared with the genetic algorithm that uses functional aggregation for all the same VNFs, the load-balancing index of the GA-PSO algorithm given the same number of tasks was slightly lower than that of the GA algorithm because the GA-PSO algorithm had better optimization performance and convergence. The load-balancing index of the RANDOM algorithm was lower than the deployment when the similarity threshold was 0.3 because the RANDOM algorithm randomly deployed the VNFs in the service function chaining. Compared with aggregating all the same VNFs, the deployment of RANDOM was more random. It will not deploy all the same VNFs on the same physical node for queuing. Therefore, the load-balancing index of the RANDOM algorithm was lower than the GA-PSO deployment when the similarity threshold was 0.3. When the similarity threshold was 0.7, the load-balancing index reached the lowest value. This was because when the similarity threshold was smaller, the aggregation degree of the VNFs was higher, which caused an excessive load on the physical nodes. When the similarity threshold was higher, the aggregation degree of the VNFs was lower. The deployment of SFC mainly relies on resource consumption, and thus, the load balancing at this time was not necessarily the lowest value. Therefore, when the similarity threshold was 0.7, the load-balancing index had the lowest value.

**Figure 8:**
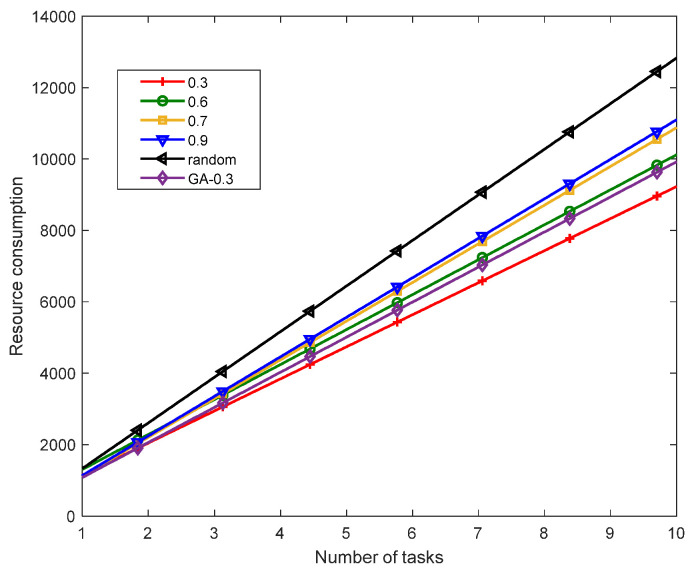
Comparison of the resource consumption with different similarity thresholds.

Figure 8 shows the resource consumption comparison of the single-chain deployment method proposed in this study with the different similarity thresholds, the GA algorithm with a similarity threshold of 0.3 and the randomly deployed RANDOM algorithm with different numbers of tasks. When the similarity threshold was 0.3, the resource consumption of the GA algorithm was higher than that of GA-PSO under the same number of tasks. When the same aggregation method was used, the GA-PSO algorithm had better optimization performance and convergence. It can be seen from the figure that as the similarity threshold gradually increased, resource consumption also increased. When the task similarity threshold gradually increased, the number of aggregated VNFs continued to decrease. As a result, more and more VNFs needed to be instantiated. The increase in instantiation overhead led to an increase in resource consumption. The RANDOM algorithm had the highest resource consumption due to its deployment without any optimization strategy.

**Figure 9:**
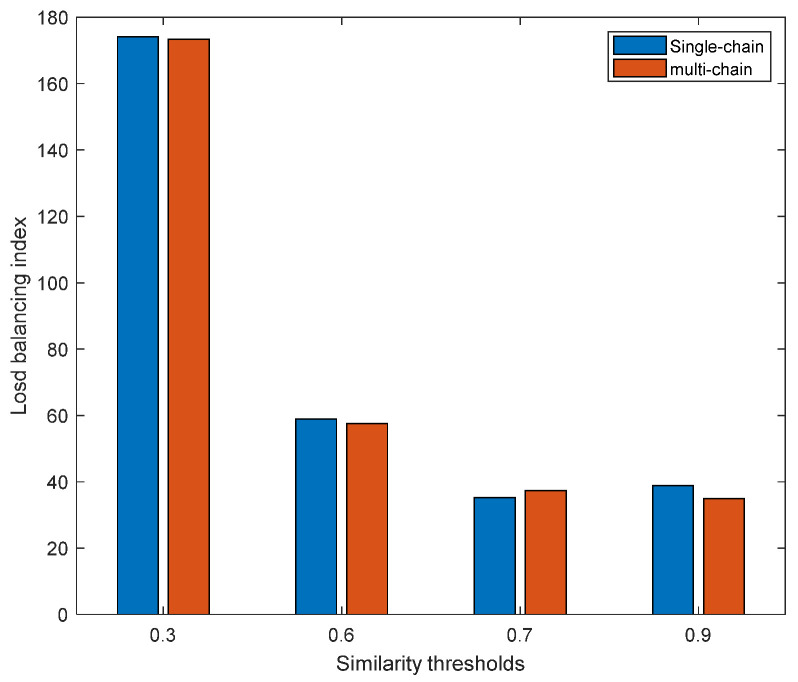
Comparison of the single-chain and multi-chain load-balancing indexes with different similarity thresholds.

Figure 9 shows the comparison of single-chain and multi-chain load-balancing indexes with different similarity thresholds. For a single chain, as the similarity threshold increased, its load-balancing index had the lowest value at 0.7. For a multi-chain, its load-balancing index always showed a downward trend. Due to the different deployment methods of single-chain and multi-chain, their changing trends were different as the similarity threshold increased. The single-chain deployment method deploys SFCs in the same category in a reference order. After each service function chaining was deployed, the subsequent SFCs implement function sharing occurred for the same VNF. As the similarity threshold increased, the deployment of each SFC depends mostly on resource consumption, resulting in that load balancing at that time not necessarily being at the lowest value, and thus, the lowest value appeared at 0.7. The multi-chain deployment method deployed SFCs in the same category simultaneously. First, the system selected the optimal deployment location of the aggregated VNF and then found the deployment location of other VNFs. As the similarity threshold increased, multiple SFCs found the optimal path together and reduced the dependence of a single SFC on resource consumption, and thus, the load-balancing index was on a downward trend. As the similarity threshold increased, except when the similarity threshold was 0.7, the load-balancing index of multi-chain deployment was slightly higher than that of a single chain. Under other similarity threshold divisions, the load-balancing index of the multi-chain system was lower than that of the single-chain system. Therefore, to reduce the load balance, multi-chain deployment was better than single-chain deployment in most cases.

**Figure 10:**
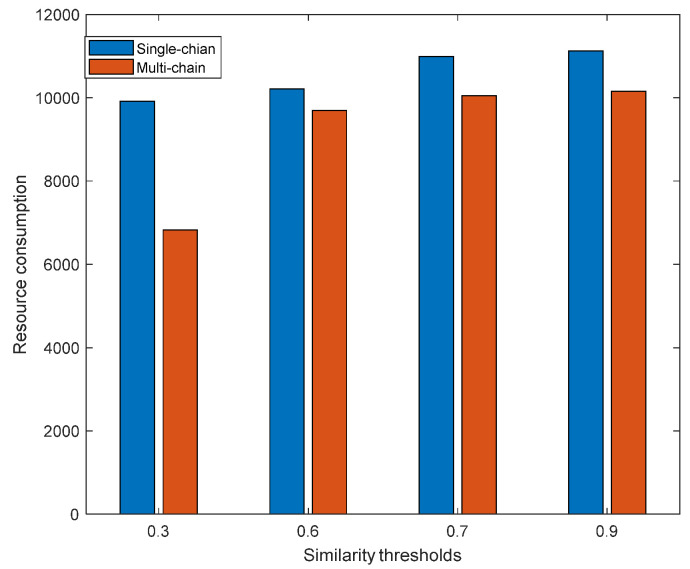
Comparison of the single-chain and multi-chain resource consumption with different similarity thresholds.

Figure 10 shows the comparison of the single-chain and multi-chain resource consumption with different similarity thresholds. It can be seen that with the same similarity threshold, the resource consumption of the multi-chain system was always lower than that of the single-chain system. Because multi-chain deployment comprehensively considers the optimal deployment location of the virtual network to be aggregated, its deployment overhead will be lower than that of single-chain deployment. Regardless of whether it is a single-chain or multi-chain system, as the similarity threshold increased, its resource consumption also gradually rose. When the task similarity threshold gradually increased, as the number of aggregated VNFs continued to decrease, more and more VNFs needed to be instantiated, resulting in an increase in the instantiation overhead and resource consumption. By comparison, with the same similarity threshold, multi-chain deployment was better than single-chain deployment for resource consumption. The similarity threshold is the primary basis for task classification. When the similarity threshold is lower, the aggregation degree of the VNFs will be higher. Although resource consumption will be reduced, it will still cause excessive load unbalancing and affect service quality. When the similarity threshold is higher, the aggregation degree of the VNF will be lower, which makes the resource consumption higher. Therefore, it is necessary to select the appropriate similarity to classify tasks according to the needs of the scene.

## 5. Conclusions

This study mainly investigated the load imbalance caused by the use of function aggregation in the deployment of SFC in the air–ground network under the network function virtualization technology. A virtual network function (VNF) aggregation scheme based on task similarity was proposed. Before SFC mapping, the air–ground network resource consumption and load imbalance were considered. A model for selecting VNFs to be aggregated based on task similarity was established. The tasks were classified according to the similarity, and the functions to be aggregated were selected within the class under the constraints of the underlying physical resources, and the load balance was achieved by adjusting the similarity threshold. We adopted the SFC mapping selection scheme based on network resource awareness and obtained the most suitable physical nodes for single-chain and multi-chain mapping according to the various attributes of physical network nodes to make full use of the underlying physical resources to reduce the resource consumption and load imbalance. It was shown that the proposed algorithm solved the load imbalance problem caused by the aggregation of functions, and the similarity threshold was adjusted to meet the demand for load balancing in different scenarios. However, the method of task classification still needs to be improved. In the future, task classification will be expanded in combination with machine learning methods to better achieve functional aggregation.

## Data Availability

Not applicable.
